# Enhanced PeriOperative Care and Health protection programme for the prevention of surgical site infections after elective abdominal surgery (EPO_2_CH): statistical analysis plan of a randomised controlled multicentre superiority trial

**DOI:** 10.1186/s13063-021-05202-y

**Published:** 2021-04-21

**Authors:** S. W. de Jonge, N. Wolfhagen, A. H. Zwinderman, M. W. Hollmann, M. A. Boermeester, M. G. W. Dijkgraaf, for the EPO2CH study group, Q. J. J. Boldingh, W. J. Bom, A. Demirkiran, O. E. van Geffen, E. R. Hendriks, J. P. Hering, J. A. B. van der Hoeven, E. B. Kluyver, B. M. F. van der Leeuw, L. R. C. W. van Lonkhuijzen, L. M. Posthuma, D. J. A. Sonneveld, J. C. G. Scheijmans

**Affiliations:** 1 Department of Surgery, Amsterdam UMC, location AMC, Amsterdam Gastroenterology & Metabolism, University of Amsterdam, Amsterdam, The Netherlands; 2 Department of Epidemiology and Data Science, Amsterdam UMC, Amsterdam Public Health, University of Amsterdam, PO Box 22660, Amsterdam, 1100 DD The Netherlands; 3 Department of Anaesthesiology, Amsterdam UMC, location AMC, University of Amsterdam, Amsterdam, The Netherlands

**Keywords:** Surgical site infection, Prevention, Surgery, Anaesthesia, Cost-effectiveness

## Abstract

**Background:**

Surgical site infections (SSI) are frequent complications after elective abdominal surgery. We designed the Enhanced PeriOperative Care and Health Protection programme (EPO_2_CH) care bundle, comprising of intraoperative high fractional inspired oxygen; intraoperative goal-directed fluid therapy; active preoperative, intraoperative and postoperative warming; glucose control and treatment of hyperglycaemia (> 10 mmol L^− 1^) in diabetics as well as non-diabetics; and wound irrigation before closure using an aqueous antiseptic. We hypothesise that EPO_2_CH added to standard care reduces the incidence of SSI compared to standard care alone for elective abdominal surgery.

**Methods:**

This trial is designed as an open label, pragmatic randomised controlled parallel-group multicentre superiority trial. The primary endpoint is the incidence of SSI, defined by the Centers for Disease Control and prevention, within 30 days after surgery. The incidence of SSI is assessed using the Dutch national complication register and medical chart review. Secondary endpoints include the SSI incidence within 90 days, incidence of anastomotic leakage at 30 and 90 days, the incidence of incisional hernia within 1 year, mortality within 1 year and 5 years, quality of life, health and disability, and cost-effectiveness. Primarily, an intention-to-treat analysis will be performed to estimate the relative risk using a log binomial model. If not feasible, a logistic regression will be used to estimate the odds ratio. A per-protocol analysis will also be performed. Furthermore, the attributive effect of the distinct interventions will be explored.

**Discussion:**

The results of the EPO_2_CH trial will determine if the EPO_2_CH bundle is effective to prevent SSI incidence for patients undergoing elective abdominal surgery. Details of the statistical analysis are described in this Statistical Analysis Plan (SAP).

**Trial registration:**

Registration number: Dutch Trial Register Trial NL5572. Registered on March 3, 2016.

SAP version: V1.0, January 8, 2020. This SAP has been written based on study protocol V10.

**Supplementary Information:**

The online version contains supplementary material available at 10.1186/s13063-021-05202-y.

## Introduction

Surgical site infections (SSI) are a frequent cause of postoperative morbidity, mortality, prolonged hospital stay and excess healthcare costs [1]. Up to 9.4% of patients undergoing gastrointestinal surgery in high-income countries suffer from SSI [2]. The development of two new guidelines on SSI prevention highlighted that perioperative care can be optimised beyond current standards of care in the Netherlands [3–6]. We selected a limited set of cheap and evidence-based interventions from these guidelines and formulated an Enhanced PeriOperative Care and Health Protection programme (EPO_2_CH). The EPO_2_CH bundle comprises a bundle of interventions including intraoperative high fractional inspired oxygen (FiO_2_), intraoperative goal-directed fluid therapy (GDFT), active preoperative, intraoperative and postoperative warming, intensive glucose control and treatment of hyperglycaemia (> 10 mmol L^− 1^) in diabetics as well as non-diabetics, and wound irrigation before closure using an aqueous antiseptic. In Dutch hospitals, these interventions are readily available without further requirements to the care team.

The EPO_2_CH trial is designed to compare the effect of the EPO_2_CH bundle added to standard care compared to standard care alone on the incidence of SSI within 30 days after elective abdominal surgery with abdominal incisions larger than 5 cm. We hypothesise that adherence to the complete EPO_2_CH bundle will reduce the incidence of SSI. Secondly, we hypothesise that the distinct interventions also potentially reduce the incidence of SSI. The EPO_2_CH trial is registered with the Dutch trial register (Nederlands Trial Register, NTR) as trial NL5572 (NTR5694). The Amsterdam UMC Medical Ethics Committee (MEC) approved the study protocol. A trial protocol has been published [7]. Here we report the Statistical Analysis Plan (SAP) according to recently published guidelines [8]. This SAP has been written before outcome data were available.

## Study methods

### Study design

The EPO_2_CH trial was designed as an open label pragmatic, randomised controlled parallel-group multicentre superiority trial that compares EPO_2_CH bundle added to standard care to standard care alone. A pragmatic approach with minimal requirements for standard care was chosen to optimise feasibility and external validity.

Central random treatment allocation of operation days to either the intervention or control group was performed. Days were randomised in a 1:1 ratio according to variable block sizes, stratified per trial site. We stratified randomisation per trial site to account for potential confounding by local variability in prognostic factors, co-interventions or other unknowns related to differences between trial sites. Randomisation per day was performed to limit possible contamination between consecutive procedures on the same day. Although the number of patients may differ per day, this was assumed unrelated to treatment allocation and we assumed the number of patients at the end of the trial to be similar for both groups. Randomisation was performed using Castor EDC, an internet-based automated assignment system [9]. A total of 3000 patients, 1500 to be assigned to each group, was required according to the sample size calculation. Further details regarding the sample size calculation are published in the study protocol [7]. The Data Safety Monitoring Board (DSMB) will perform a sample size recalculation after follow-up of 1500 patients to determine the definite sample size. For the sample size recalculation, the DSMB will test the sample size assumptions in the control group of these 1500 patients after 30 days follow-up. The DSMB will also regularly assess patient safety and may advise to stop the study prematurely at its own discretion if this serves the interest of the trial participants. There will be no interim analysis for the treatment effect.

### Outcomes

Primary outcome was the incidence of SSI within 30 days after the index operation as defined by the Centers for Disease Control and prevention (CDC) [10]. The incidence was assessed using the Dutch “Landelijke Heelkundige Complicatie Registratie” (LHCR), which is the Dutch national surgical complication register, and medical chart review by the research physicians of the trial management team. If SSI in a patient met criteria of multiple classes of SSI, the most severe SSI class was scored.

Main secondary outcomes include the SSI incidence within 90 days, incidence of anastomotic leakage at 30 and 90 days, length of hospital stay, hospital readmission rate, health and disability (WHO disability assessment schedule (WHODAS) 2.0), health utility and QALY (EuroQol (EQ)-5D-3L) and related costs (institute for Medical Technology Assessment’s (iMTA) Medical Consumption Questionnaire (iMCQ) and Productivity Cost Questionnaire (iPCQ) and out of pocket expenses) [11–13]. Other (long-term) secondary outcomes include SSI incidence within 30 days assessed through a post-discharge self-assessment questionnaire (a Dutch translation of the Bluebelle Wound Healing questionnaire), SSI incidence within 30 days by self-reported wound photos, the incidence of incisional hernia within 1 year, and all-cause mortality within 1 year and 5 years [14]. Further details regarding outcomes and method of data collection are described in the study protocol [7].

### Timing of outcome assessments and analysis

Outcomes will be described and measured during routine clinical care at regular follow-up visits by the caring clinician. Typically, these follow-up visits occur 2 weeks after discharge and within 30 days after surgery. In addition, wounds are inspected at the hospital whenever symptoms of complications occur. Questionnaires will be sent at 10 days (EQ-5D-3L and wound photo), 30 days (EQ-5D-3L, wound photo, WHODAS 2.0, post-discharge self-assessment questionnaire), 60 days (EQ-5D-3L, WHODAS 2.0) and 90 days (EQ-5D-3L, WHODAS 2.0, iMCQ, iPCQ) after surgery.

The analyses and reports will be stratified by planned follow-up duration and outcome: 90 days for short-term clinical outcomes, 90 days for economic outcomes and 1 year and 5 years for long-term outcomes. For each stratum, analysis will be performed after follow-up of the last included patient is complete and data are cleaned and verified.

### Sub-study

In a sub-study of 48 patients, we will study perioperative subcutaneous oxygen pressure (PtO_2_) (mmHg), mitochondrial PtO_2_ (mmHg) and perioperative immune response. Further details regarding this sub-study are described in the study protocol [7].

## Statistical analysis

### Confidence intervals and *p* values

Statistical tests will be two-sided and a *p* value of < 0.05 will indicate statistical significance for the primary outcome. The effect of individual interventions of the bundle follow directly from our hypothesis and therefore no adjustment for multiple testing is planned. Also, no adjustment is planned for the cost-effectiveness analysis. All other analyses, including interventions outside the bundle, are considered supportive and no statistical testing will be performed. In all analyses, statistical uncertainties are expressed in 95% two-sided confidence intervals (CIs).

### Adherence and protocol deviations

Compliance to the intervention bundle is defined as complete compliance to each distinct intervention in the bundle. Compliance is measured through postoperative surveys and relevant process measures. After every procedure both the surgeon and the anaesthetist will complete a postoperative survey to describe which care, specified per intervention, was given. In addition, data from process measures for 3 of the 5 interventions (intraoperative FiO_2_, core temperature and blood glucose) are collected from the (electronic) medical record. As a measure of reliability one objectively verifiable variable will be cross-checked between survey response and process measure. Reliability will be expressed as a proportion of true responses among all responses.

In the intervention group, compliance to FiO_2_^1^ will be defined as a FiO_2_ of 0.80 ± 0.05 for at least 75% of the ventilation time. In the control group, a FiO_2_ smaller than 0.40 with a margin of 0.05, for 75% is considered treatment according to protocol. Patients requiring more oxygen for medical reasons, for example to maintain adequate saturation, after initial ventilation with an FiO_2_ of 0.45 are exempted and not considered a protocol deviation. Compliance to perioperative normothermia will be defined as core temperature above 36.5 °C, with a margin of 0.5 °C, from intubation until 1 h postoperatively. To account for incidental temperature mismeasurement (e.g. temporarily luxated temperature probe), only drops in core temperature below 36.0 °C for more than 15 min during the intraoperative phase, when temperature is measured (semi)-continuously, will be considered a protocol deviation in the intervention group. In the pre- and postoperative phase, when temperature is measured by point measurements - not (semi)-continuous probe measurement, it will be assumed that the point measurements are representative for the entire pre- or postoperative phase. In the control group, temperature management is left at the discretion of the anaesthetist and no protocol deviation is defined. Compliance to normoglycaemia is defined for all patients, diabetics as well as non-diabetics, as perioperative blood glucose levels < 10 mmol L^− 1^ or > 10 mmol L^− 1^ with adequate treatment, measured every hour during surgery or at least twice for procedures lasting more than 2 h, and once at the recovery, at day one and day two postoperatively. Patients, in the control group, typically do not receive perioperative glucose measurements unless medical conditions such as diabetes require these. Patients requiring regular measurement of blood glucose levels and/or treatment for hyperglycaemia for medical reasons will be considered to have received treatment according to protocol. No accurate process measures for GDFT and wound irrigation will be available. Due to the pragmatic nature of the trial, all other aspects of perioperative care are left at the discretion of the caring surgeon, anaesthetist and nurses.

When both the postoperative survey and the process measure are available but their outcomes are discrepant, one may overrule the other. Compliance according to recorded process measures will overrule results of postoperative surveys. However, if blood glucose levels are registered in the postoperative survey but not recorded in the medical record, we assume these are measured at the point of care without registration in the medical record. Compliance according to the postoperative survey will then overrule the process measure.

Randomised patients who do not meet eligibility criteria based on preoperative criteria or an intraoperative change in treatment plan that would render participants not at risk, or at an unrepresentative risk of the outcome are considered wrongful randomisations. Examples include a change of the surgical plan that results in abdominal wound(s) smaller than 5 cm (e.g. laparoscopic surgery involving only trocar openings or a transanal approach). This will result in exclusion from the trial and the participant will be replaced. Post-randomisation exclusion can be performed by the trial management team (TMT) or by any of the local investigators. Although not blinded, exclusion will be done as soon as possible after surgery and will be independent of the study outcome.

### Analysis populations

**Fig. 1:**
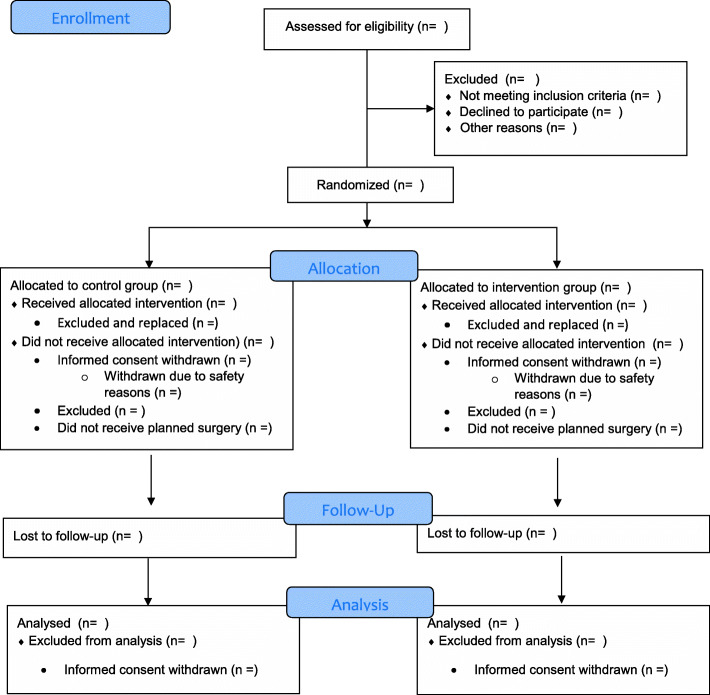
“CONSORT Flow diagram EPO_2_CH Trial”

Three analyses populations are defined. The *intention-to-treat* population will include all trial participants according to their treatment allocation independent of compliance. Figure 1 will depict the intention-to-treat population. The *per-protocol* population will include participants, of whom compliance to the complete EPO_2_CH bundle is known from postoperative surveys and/or process measures. If postoperative survey results are missing, compliance to the complete bundle is unknown and patients will be excluded from the per-protocol population. The *safety* population will include all participants and postoperatively excluded patients according to the receipt of none, or one or more components of the bundle based on postoperative surveys and/or process measures. When both postoperative survey results and process measures are missing for all of the interventions, adherence is unknown and patients will be reported separately.

## Trial population

### Screening, eligibility, recruitment and follow-up

Adult patients, scheduled for elective abdominal surgery involving an abdominal incision larger than 5 cm (open surgery as well as laparoscopic surgery with incisions larger than 5 cm (hand-assisted and/or for the excision specimen)), are eligible. Exclusion criteria are described in the study protocol [7]. Number of patients screened, ineligible, eligible and recruited, and eligible and not recruited are provided with the corresponding reasons. Given the wide scope and limited resources, it may be challenging to reliably track the exact number of patients screened. If hospitals will not be able to reproduce the number of patients screened, hospitals will be asked to make an approximation, preferably using insurance data. To ensure independence of observations, patients may only participate once in the trial, regardless of fulfilment of in- and exclusion criteria for any potential future operation. After consent or treatment allocation, participants may prove to be ineligible and will be replaced. Numbers and reasons for ineligibility will be provided. For the clinical primary outcome, we assume that patients either contact the hospital in case of SSI or mention it in any postoperative contact. Patients are regarded lost to follow-up if there is no registered postoperative contact with the hospital within the corresponding follow-up period. For secondary outcomes such as health and disability, health utility, QALY and costs, lost to follow-up will be regarded if patients, despite multiple efforts, do not return questionnaires. The last returned questionnaire will be the last moment of follow-up.

The Consolidated Standards of Reporting Trials (CONSORT) flow diagram, shown in Fig. 1, will depict the number of participants ineligible, eligible, consented, randomised, received allocated treatment, excluded, replaced, withdrawn and lost to follow-up for the primary outcome. The level and timing of exclusion, withdrawal or lost to follow-up will also be provided. A flow diagram for the cost-effectiveness is described in Additional file 1: Appendix 1.

### Baseline characteristics

The baseline characteristics will be reported separate per treatment arm, as demonstrated in Table 1. The table includes sex (% male), age (years), BMI (m^2^/kg), ASA score following ASA Physical Classification system (%), smoking (%), (insulin-dependent) diabetes mellitus (% yes), cardiovascular heart diseases other than hypertension (% yes), chronic obstructive pulmonary disease (% yes), type of centre (academic, top-clinical, general hospital), indication for surgery (benign/malignancy) and surgery type (upper gastrointestinal/hepato-pancreato-biliary/colorectal/general/gynaecologic surgery). Categorical data will be described by counts and percentages. Continuous data will be described as mean with standard deviation if normally distributed, and median and interquartile range if not.

**Table 1:** Baseline characteristics

Characteristic	Intervention group (*n* =)	Control group (*n* =)
Sex (% male)		
Age (years)		
BMI (m^2^/kg)		
Physical status score		
ASA I		
ASA II		
ASA III		
ASA IV		
Smoking (%)		
Diabetes mellitus (%)		
Insulin dependent		
Non-insulin dependent		
Cardiovascular disease other than hypertension (%)		
Chronic Obstructive Pulmonary Disease (COPD) (%)		
Type of hospital (%)		
Academic		
Top-clinical		
General		
Indication for surgery (%)		
Benign		
Malignancy		
Surgery type (%)		
Upper gastrointestinal surgery		
Hepato-pancreato-biliary surgery		
Colorectal surgery		
General surgery		
Gynaecologic surgery		

### Perioperative procedure characteristics

The perioperative procedure characteristics will be reported separately per treatment arm, as demonstrated in Table 2. The table includes preoperative antibiotics (%), mean arterial blood pressure, mean heart rate, average core temperature in Celsius, average FiO_2_, average oxygen saturation, partial pressure of arterial oxygen, subcutaneous oxygen tension, estimated blood loss, crystalloid (ml), colloid (ml), blood products (units), vasopressors, wound classification according to CDC (clean/clean-contaminated/contaminated/dirty) [15], operation time in minutes, local anaesthesia (yes/no), pain score (area under the curve in first 3 days) and highest pain score on first postoperative day. Categorical data will be described by counts and percentages. Continuous data will be described as mean with standard deviation if normally distributed, and median and interquartile range if not.

**Table 2:** Perioperative procedure characteristics

Characteristic	Intervention group (*n* =)	Control group (*n* =)
Preoperative antibiotics (%)		
Within 15–60 min before incision (%)		
Arterial blood pressure, mmHg		
Heart rate, bpm		
Core temperature, C		
Inspired oxygen fraction		
Oxygen saturation		
Partial pressure of arterial oxygen		
Subcutaneous oxygen tension		
Estimated blood loss, ml		
Crystalloid, ml		
Colloid, ml		
Blood products, units		
Patients with blood products, *n*		
Vasopressors, mg		
Wound classification (%)		
Clean		
Clean/contaminated		
Contaminated		
Dirty		
Operation time (minutes)		
Epidural anaesthesia		
Pain (VAS)		
Highest pain score POD1		
Cumulative score within first 3 days postoperative		

*POD* postoperative day, *VAS* visual analogue scale

The number and percentage of participants compliant according to each survey item or corresponding process measure when available, and the number and percentage of participants compliant to 0, 1, 2, 3, 4 or all 5 interventions will be presented by the treatment group as demonstrated in Table 3.

**Table 3:** Compliance to the interventions of the EPO_2_CH bundle

Intervention	Intervention group (*n* =)	Control group (*n* =)
Normothermia (%)		
Missing (%)		
Goal-directed fluid therapy (%)		
Missing (%)		
Hyperoxygenation (%)		
Missing (%)		
Normoglycemia (%)		
Missing (%)		
Wound irrigation (%)		
Missing (%)		
Compliant to 0 (%)		
Compliant to 1 (%)		
Compliant to 2 (%)		
Compliant to 3 (%)		
Compliant to 4 (%)		
Compliant to 5 (%)		
Missing (%)		

## Analysis

### Analysis methods

Log binomial regression will be used to estimate relative risks and corresponding CIs for binary outcomes. If the model fails to converge, logistic regression will be used instead. The estimated odds ratio (OR) and corresponding CI will be used for statistical interpretation and a recalculated relative risk for point estimate interpretation. Linear regression will be used to estimate relative risks and corresponding confidence intervals for continuous outcome data. Quality of life data will be analysed as repeated measurement using linear mixed modelling. To account for the stratified randomisation by trial site and independence of observations, a covariate for trial site will be included in the models. If some strata are small (smaller than 50 participants), trial sites will be clustered according to hospital type (academic, top-clinical/regional teaching and general) as a proxy based on similarities in prognostic factors and potential co-interventions. In the primary analysis, all outcomes will be analysed according to intention-to-treat. Short term clinical outcomes will be reported as demonstrated in Table 4. Long-term and health economic outcomes will be reported in separate publications.

**Table 4:** Primary and secondary outcomes

Characteristic	Intervention group (*n* =)	Control group (*n* =)	Relative risk (95% CI) ITT	Corrected relative risk (95% CI) PP	Corrected relative risk (95% CI) HP
Surgical site infections incidence within 30 days					
Surgical site infections incidence within 90 days					
Anastomotic leakage incidence within 30 days					
Length of stay, median (IQR), d					
Readmissions, median no. per patients (IQR)					
Patients with Serious Adverse Events, no (%)					
Serious adverse events, no					
Clavien Dindo III					
Clavien Dindo IV					
Clavien Dindo V					
Quality adjusted life years					

*ITT* intention to treat population, *IQR* interquartile range+, *PPP* per-protocol population, *HP* harms population

### Sensitivity and additional analyses

Randomisation of operation days per trial site enables within-centre comparison of treatments and increases power. A sensitivity analysis of the primary outcome will be conducted that accounts for within-centre comparison of treatments according to the effect estimate (Additional file 1: Appendix 2). This will be done with a log binomial generalised estimating equation (GEE) or the log Poisson GEE, if the former fails to converge. If major test assumptions are met and the hypothesis test of this analysis diverges from the primary analysis, all further analyses will include within-centre comparisons in parallel.

All outcomes will also be analysed according to the per-protocol population after adjustment for confounding factors due to incomplete adherence to the assigned treatments or use of off-protocol concomitant therapies. Variables will be considered for adjustment based on the criterion for confounder selection by VanderWeele and Shpitser and include preoperative body mass index, insulin dependent diabetes mellitus, cardiovascular diseases other than hypertension, chronic obstructive pulmonary disease and any other variables that meet these criteria and pass statistical variable selection [16, 17]. Procedure duration will also be considered for adjustment despite being measured during the exposure. Procedure duration is considered an important proxy for the complexity of the procedure. We assume that the interventions comprising the EPO_2_CH bundle do not prolong the procedure duration. This approach will also be applied to analyses of serious adverse events and mortality according to the safety population.

We will also explore the attributive effect of the distinct interventions and a potential dose response effect. To limit loss of information, this analysis will be conducted on patients from both the control and intervention group. Using the before mentioned model building strategy, we will built two models. The first will include separate exposure variables for each of the distinct interventions. Because previous studies suggest high FiO_2_, GDFT, normothermia and glucose levels may interact, [18–20] two- and three-way interactions between the distinct elements of the bundle will also be explored. The second model will include a single ordinal exposure variable with six levels for adhering to 0, 1, 2, 3, 4 or all 5 of the bundle interventions. The Cochrane Armitage test will be used to test for a potential dose response relationship.

Furthermore, an additional analysis for individual attributive effects will be conducted applying the same approach to distinct components of the Dutch [PostOperative Wound Infection] (POWI) protocol and promising innovative interventions outside the bundle under investigation. The POWI protocol comprises hygiene discipline (measured by number of operating theatre door movements), timing of antibiotic prophylaxis (between 15 and 60 min prior to incision), normothermia (aimed at a core temperature of 36.5 °C postoperatively), and the avoidance of preoperative hair removal or use of clipper but not razor [21].

If possible, we will perform internal verification of (a sample of) the primary outcome compared to secondary outcomes such as postoperative questionnaire and postoperative wound photos. Furthermore, a time series analysis of the given standard care in the control group will be performed to analyse if the standard care stayed the same during the study period. Typical standard care is described in the trial protocol [7]. All interventions of the EPO_2_CH bundle will be analysed separately. The time series will constitute of groups of 200 consecutive patients. Interventions will be operationalised as previously described. Dichotomous variables will be analysed using Pearson’s chi^2^ or Fisher’s exact test, as appropriate. Continuous variables will be analysed using ANOVA or Jonckheere-Terpstra test, as appropriate.

## Economic evaluation

### Cost effectiveness analysis

Economic evaluation of the EPO_2_CH bundle compared to standard care will be performed as cost-effectiveness and cost-utility analyses from a societal perspective with the costs per patient without SSI and the costs per quality adjusted life year (QALY) as primary economic outcomes respectively. The time horizon will be 3 months. Considering this time horizon, no discounting of effects and costs will be done. Incremental cost-effectiveness ratios will be calculated as the extra costs per additional patient without SSI and as the extra costs per QALY gained. Sampling variability will be accounted for by bias-corrected and accelerated non-parametric bootstrapping [22]. Results will be displayed graphically with cost-effectiveness planes and cost-effectiveness acceptability curves for societal willingness-to-pay levels per QALY up to 30,000 euros. One-way and multi-way sensitivity analyses will be done for the unit costs in Euros of the EPO_2_CH bundle and for sex- and age-specific rather than general unit costs of productivity loss. Subgroup analyses will be done for (i) patients with upper versus lower gastro-intestinal tract procedures, (ii) surgical procedures above versus below the median duration, and (iii) clean versus clean-contaminated procedures to account for differences in baseline risk of SSI. A scenario analysis for a delineated EPO_2_CH bundle will be applied, if compliance data from the self-score checklists for surgeons and anaesthetists and/or the process measure indicate that the components of the bundle vary by their contribution to the overall effect on SSI. A second scenario analysis will be run to reflect an international perspective by applying utility weights from the UK [23] respectively USA [24] to the observed health states and by applying purchasing power parities from the Organisation of Economic Co-operation and Development to transpose the observed costs in pound sterling respectively US dollar equivalents [25].

Medical, patient and employer costs will be included in the evaluation. The medical costs cover the costs of surgery, anaesthesia, theatre, perioperative materials, inpatient stay on the ICU and the wards, diagnostic and therapeutic (other than surgical) procedures, and medication against infections. The patient costs include out-of-pocket expenses like over-the-counter medication and health care-related travel. The employer costs reflect losses of productivity resulting from absenteeism and presenteeism. Unit costing of health care resources will be derived from the most recent guidelines on national health care costing for (pharmaco-) economic evaluations at the time of analysis [13]. Market prices will be used for medications. Productivity losses will be based on the friction cost method (with general as well as age- and sex-specific unit costs per hour of productivity loss), with the most recent estimate at the time of analysis for the length of the friction period as reference. All costs will be expressed in Euro for the base year 2019. Costs borne in other calendar years will be price indexed (based on general yearly consumer price indices from Statistics Netherlands [26]). Costs will be calculated for individual patients as the product sum of actual resource use and the respective unit costs.

EQ-5D-3L scoring profiles will be converted into a health utility score based on Dutch general population-based tariffs of time trade-off ratings of health states [27]. QALYs will be calculated for each patient (maximum 0.25 per patient) after linear interpolation between the successive health utility assessment over time. In the absence of a baseline measurement, we will use the value of day 10 for the preceding 9 days and apply interpolation between days 10 till 90.

### Budget impact analysis

The short- and mid-term budget impact of the EPO_2_CH enhanced perioperative care programme will be assessed from governmental, insurer and provider perspectives in accordance with the ISPOR guidelines [28]. The analysis will be prevalence based, reflecting the net savings of fewer SSI during the years when the optimised surgical procedures actually take place. Assessment is episodic; savings will be quantified per abdominal procedure. A next abdominal surgical procedure for the same patient after the follow-up period of 3 months will be counted as a new incident case. The time horizon of the budget impact is 4 years, starting in 2022. Projected numbers of indicated abdominal surgical procedures will be derived from historical health care registry data (www.opendisdata.nl) and curve fitting, selecting the best fitting model of lowest order to temper over- or underestimations at the end of the time horizon. Several EPO_2_CH bundle diffusion scenarios will be off-set against the base case scenario of using standard operative care. Full (100%) and partial (75%, 50%) implementation scenarios will be explored in combination with immediate implementation (100% of full/partial level (FPL) in 2022) or gradual (30% of FPL in 2022, 60% of FPL in 2023, 100% of FPL in 2024 and 2025). Depending on the publication date, the time period 2023–2026 may be used for reference. Sensitivity analyses will be performed to account for the imprecision of SSI rate reductions and their associated impact on health care reimbursement. Depending on the results from the subgroup analyses and from the delineation scenario mentioned under the cost-effectiveness analysis (see the previous section), additional scenarios with alternative EPO_2_CH bundle implementation targets may become opportune and if so, will be quantified.

The most recent guidelines for (unit) costing in health care research will be applied [13]. In case of impact assessments concerning premium financed health care from the health care insurer perspective, the most recent Dutch national tariffs for diagnosis-treatment combinations available at the time of analysis will be used.

### Missing data

In case of missing data of clinician-reported outcomes and mortality, participants will be considered lost to follow-up for the respective outcome. We assume this to be unrelated to the interventions and a complete case analysis will be conducted. This approach will also be applied to SSI according to the post discharge self-assessment questionnaire. Potential missing data of patient-reported outcomes for the economic evaluation will be assumed to be at random and dealt with by multivariate imputation by chained equations (MICE) [29, 30]. The coefficients of five rounds of imputation, based on relevant predictors for each respective questionnaire separately, will be combined to obtain final estimates of the missing value. Variables selected for prediction include: before mentioned baseline characteristics, treatment arm, SSI occurrence, length of hospital stay and the non-missing questionnaires. Percentage of missing data will be reported. A sensitivity analysis will be conducted using a complete case analysis. Missing values of baseline characteristics will not be imputed. When displaying the baseline characteristics, the actual denominator will be stated for dichotomous variables. For continuous variables, a footnote to show the number of patients for whom the variable was available will be stated.

### Harms

Harms will be reported in accordance with the checklist of the CONSORT Group [31]. Serious adverse events (SAE) are defined as any event that is fatal, threatens the life of the subject, makes hospital admission or an extension of the admission necessary, causes persistent or significant invalidity or work disability, manifests itself in a congenital abnormality or malformation and/or could, according to the trial management team (TMT), have developed to a serious undesired medical event, but was, however, prevented due to premature interference [32]. Due to the heterogeneity of the interventions, we report all SAE. SAE have to be reported to the TMT by the participating trial sites. The TMT reviews all individual medical records of participants to check SAE. SAE will be categorised according to the Clavien Dindo [33]. The number of SAE and number of participants with SAE will be reported per treatment arm. Poisson regression, negative binomial regression or their zero-inflated alternatives will be applied to test for differences between treatments in the number of SAEs, depending on the best data fit. Chi-square testing will be done for the association between treatment and experiencing at least one SAE.

### Software

For the analyses of the EPO_2_CH trial R version 3.6.1. and SPSS Statistics version 24 will be used.

## Discussion

The EPO_2_CH study assesses the effect of a care bundle, consisting of intraoperative high FiO_2_, GDFT, active pre- intra- and postoperative warming, intensive glucose control with treatment of hyperglycaemia (> 10 mmol L^− 1^) and wound irrigation, compared to standard care to prevent SSI. This SAP describes the intended analyses of data collected throughout the study upon completion of follow-up of the respective outcomes. The results will be shared through publications in peer-reviewed medical journals in strata according to outcome specific follow-up when data collection and analyses are completed. By publication of this SAP, we provide transparency, allowing timely comments and suggestions for modifications or additional analyses.

The trial hypothesis is that the EPO_2_CH bundle is superior compared to standard care alone for the prevention of SSI. SSI are common complications with a reported incidence of 9.4% in patients after gastrointestinal surgery in high-income countries [2]. It is expected that, using evidence-based strategies, many are preventable [6]. So far, many individual interventions have been studied but with limited success. We chose to combine several evidence-based strategies into a bundle to further improve clinical outcomes.

We chose a pragmatic design with minimal requirements for standard care to optimise feasibility of the study and external validity of its results. This approach also has limitations. While we expect that practice variation outside the bundle under investigation will randomise evenly, (partial) adoption of interventions in the control group leads to contamination between groups and biases the results towards the null hypothesis. Potential gaps in compliance in the intervention group and contamination between groups may also occur despite precautions, and further aggravate this effect. As a result, the effect estimated in the primary analysis on the intention to treat population may underestimate what is maximally achievable by implementation of those interventions. Because the expected effect size affects the statistical power, we accounted for some contamination between groups in our sample size calculation by choosing a smaller expected relative risk reduction then may be expected based on the available evidence [6]. In addition, we used a conservative estimate of SSI risk in the control group for our sample size calculations to prevent underestimation. However, it remains to be determined whether these measures prove insufficient to overcome the observed contamination and SSI incidence. We will take this into account when interpreting the final effect estimate. To ensure that we will get an impression of the maximally achievable effect, an additional analysis will be conducted on the per-protocol population after adjustment for confounding factors. A similar approach is used to estimate potential harms in the safety population. Compliance and practice variation will be measured using postoperative surveys and carefully defined process measures based on minute to minute data. Any degree of practice variation or contamination that will be found will also be used to explore the attributive effect of existing infection prevention measures, the distinct components of the intervention bundle and other promising interventions outside of the bundle.

No follow up visits are required in addition to routine clinical follow up. While this may lead to some undetected SSI, we expect this is unrelated to the intervention and will evenly distribute across the two study arms. Importantly, the data on SSI incidence used for our sample size calculation was also derived from routine clinical follow-up and should be representative for this approach.

To enhance interpretability of the effect estimate, we choose to calculate relative risks using a log binomial model instead of odds ratios with ordinary logistic regression. We limited covariate adjustment to the stratification variables during randomisation and ignored potential gains in statistical power through estimation of within-centre effects or further covariate adjustment to keep our primary analysis simple and transparent. We will test the robustness of the effect estimate against these decisions with sensitivity analyses.

We expect that the results of the EPO_2_CH trial, analysed according to the SAP presented in this manuscript, will answer our primary research question; does the EPO_2_CH bundle reduce the risk of SSI when compared to standard care. Additional analyses will help gain further understanding of the economic effects and the attributive effects of individual components of the bundle and other interventions outside the bundle under investigation. These results will reveal the potential gains of implementation of important components of recent SSI prevention guidelines in the Netherlands and other comparable high-income countries [3–5].

## Supplementary Information

**Additional file 1: Appendix 1.** Additional figures. **Appendix 2.** Theoretical considerations: Elaboration on within-centre effect. **Appendix 3.** Completed checklist for Guideline for Statistical Analysis Plan. **Appendix 4.** Signature sheet.

**Additional file 2.**

## Data Availability

SW, NW and MB will have access to the Data Management Plan, Trial Master File and Standard Operating Procedures (SOPs). SW, NW, MD, MWH and MB will have access to the complete dataset. Local principal investigators will have access to relevant SOPs, Investigation Site File and data of patients enrolled in their centre. Availability of data and materials for this publication is not applicable.

## Abbreviations

ASA score: ASA Physical Classification system
BMI: Body mass index
bpm: Beats per minute
C: Celsius
CDC: Centers for Disease Control and prevention
CIs: Confidence intervals
COPD: Chronic Obstructive Pulmonary Disease
CONSORT: Consolidated Standards of Reporting Trials
DSMB: Data Safety Monitoring Board
EPO_2_CH: Enhanced PeriOperative Care and Health Protection programme
EQ-5D: EuroQol-5D
FeO_2_: Fraction expired oxygen
FiO_2_: Fractional inspired oxygen
FPL: Full/partial level
GEE: Generalised estimating equation
GDFT: Goal-directed fluid therapy
HP: Harms population
IC: Intensive care unit
iMCQ: iMTA Medical Consumption Questionnaire
iMTA: Institute for Medical Technology Assessment
iPCQ: iMTA Productivity Cost Questionnaire
IQR: Interquartile range
LHCR: Landelijke Heelkundige Complicatie Registratie
MEC: Medical Ethics Committee
MICE: Multivariate imputation by chained equations
NTR: Nederlands Trial Register
OR: Odds ratio
POD: Postoperative day
POWI: PostOperative Wound Infection
PP: Per-protocol
PtO_2_: Oxygen pressure
QALY: Quality adjusted life year
SAE: Serious adverse events
SAP: Statistical Analysis Plan
SOP: Standard Operating Procedure
SSI: Surgical site infections
TMT: Trial management team
VAS: Visual analogue scale
WHO: World Health Organization
WHODAS: WHO disability assessment schedule

## Footnotes

1 If fraction expired oxygen (FeO_2_) is measured, the reference is a FeO_2_ of 0.75 ± 0.05 for the intervention group and a FeO_2_ of smaller than 0.40 for the control group.
