# Supplementary material for: Enhanced PeriOperative Care and Health protection programme for the prevention of surgical site infections after elective abdominal surgery (EPO2CH): statistical analysis plan of a randomised controlled multicentre superiority trial
Source: Trials. 2021 Apr 21;22:297. doi: 10.1186/s13063-021-05202-y (PMC8059309; doi:10.1186/s13063-021-05202-y)
Supplement: Supplementary file 1 — Additional file 1: Appendix 1. Additional figures. Appendix 2. Theoretical considerations: Elaboration on within-centre effect. Appendix 3. Completed checklist for Guideline for Statistical Analysis Plan. Appendix 4. Signature sheet. [file 13063_2021_5202_MOESM1_ESM.pdf]

Appendix 1: Additional figures

Figure 2: “CONSORT Flow diagram” Economic evaluation

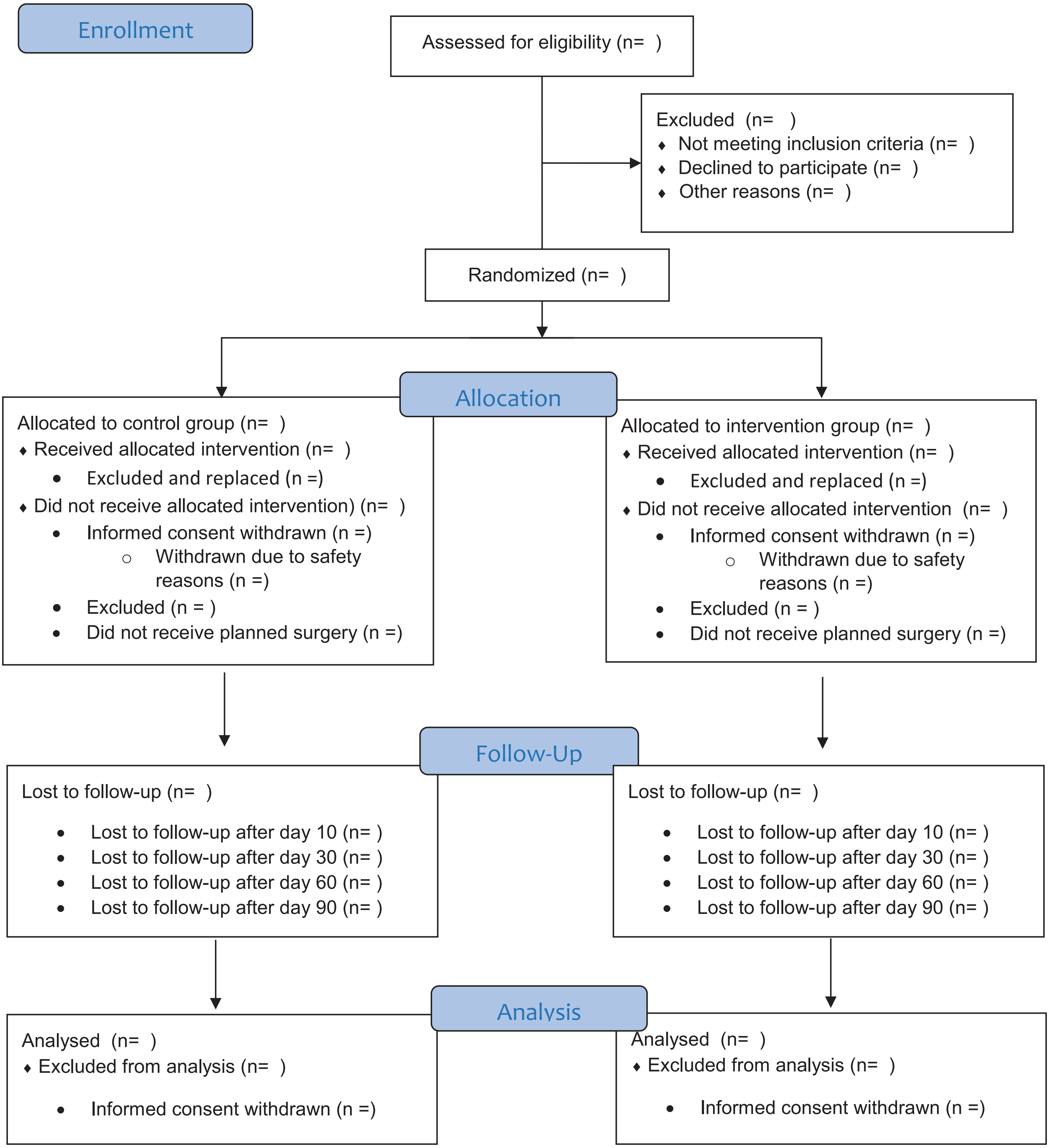

Appendix 2: Theoretical considerations: Elaboration on within-centre effect

The null hypothesis states that there is no difference between the outcome in the control group compared to the intervention group. In order to statistically test the null hypothesis, multiple statistical measures can be chosen to summarise this outcome. One possibility is to account for a within-centre effect of each participating trial site. This may increase power. In this Appendix we explore this possibility. First we describe the effect estimate and its variance. Secondly we describe the effect of a difference between the number of days a trial site randomises participants.

1. Effect estimate and its variance

   During the study period of the EPOCH trial randomisation is performed daily per participating trial site between the control group (A) and the intervention group (B) in k trials sites. The primary outcome observed will be dichotomous for all participants: $Y = 1$ or $Y = 0$. At trial site i and at day j, the number of participants will be n_{ij}. The outcome for participant ℓ will be noted as $y_{ij\ell}$.

   First we determine the mean difference of outcomes between group A and group B at k hospitals:

$$\bar{\bar{d}} = \frac{1}{k}\sum_{i}^{k} \bar{y}_i^A - \bar{y}_i^B \tag{1}$$

   Here $\bar{y}_i^A$ en $\bar{y}_i^B$ represents the 'mean' outcome of participants in hospital i in group A and group B respectively. The statistical test for the null hypothesis $H_0 : \bar{\bar{\delta}} = 0$,is a z-test following $z = \frac{\bar{\bar{d}}}{\sqrt{Var\ (\bar{\bar{d}})}}$, of which $Var\ (\bar{\bar{d}})$ is the variance of $(\bar{\bar{d}})$.

   The statistical power of the trial is the probability that $|z| > z_\alpha$. The relation between statistical power, sample size and effect $(\bar{\bar{d}})$ is represented as:

$$(z_\alpha + z_\beta) = \frac{\bar{\bar{d}}}{\sqrt{Var\ (\bar{\bar{d}})}} \tag{2}$$

   Here z_α and z_β are ordinates of a standard normal distribution with a significance of α and 1-power of β.

If we assume that the outcome of participants in the participating trials sites are independent of each other, the variance can be expressed as:

$$Var(\bar{\bar{d}}) = Var(\frac{1}{k}\sum_{i}^{k}\bar{y}_i^A - \bar{y}_i^B) = \frac{1}{k^2}\sum_{i}^{k}Var(\bar{y}_i^A - \bar{y}_i^B)$$

(3)

This can also be expressed as:

$$Var(\bar{\bar{d}}) = \frac{1}{k^2}\sum_{i}^{k}Var(\bar{y}_i^A - \bar{y}_i^B) = \frac{1}{k^2}\sum_{i}^{k}Var(\bar{y}_i^A) + Var(\bar{y}_i^B) - 2Cov(\bar{y}_i^A, \bar{y}_i^B)$$

(4)

Here $\bar{y}_i^A$ represents the mean outcome of the participants at trial site i that received treatment according to group A:

$$\bar{y}_i^A = \frac{1}{m_i}\sum_{j}^{m_i}\frac{1}{n_{ij}}\sum_{\ell}^{n_{ij}}y_{ij\ell}$$

(5)

The number of days participants are randomised in group A at trial site i is represented as m_i and n_{ij} represents the number of participants on day j at trial site i that are randomised in group A. The variance of $\bar{y}_i^A$ can be expressed as:

$$Var(\bar{y}_i^A) = Var\left(\frac{1}{m_i}\sum_{j}^{m_i}\frac{1}{n_{ij}}\sum_{\ell}^{n_{ij}}y_{ij\ell}\right) = \frac{1}{m_i^2}\,Var\left(\sum_{j}^{m_i}\frac{1}{n_{ij}}\sum_{\ell}^{n_{ij}}y_{ij\ell}\right) =$$

$$= \frac{1}{{m_i}^2}\sum_{j}^{m_i}\left(\frac{\sigma_{ij}^2}{n_{ij}}\left(1 + (n_{ij} - 1)\rho_{i1}\right) + 2\sum_{j'>j}^{m_i}\sigma_{ij}^2\rho_{i2}\right)$$

Assuming that; (i) the variance σ_{ij}^2 of the outcomes of all participants that are randomised for day j at trial site i are the same; (ii) the correlation ρ_{i1} between the outcomes of two participants that are randomised for the same day j is the same for all two participants that are randomised for the same day; iii) the correlation ρ_{i2} between outcomes of two of participants that are randomised for day n day j and j' is the equal for all groups of two participants.

It seems also reasonable to assume that (iv) variance is equal for all days ($\sigma_{ij}^2 = \sigma_i^2$). Possibly we can also assume that the number of participants that receive treatment is constant for all days ($n_{ij} = n_i$). This allows us to simplify variance of $\bar{y}_i^A$ as :

$$Var(\bar{y}_i^A) = \frac{1}{{m_i}^2}\sum_{j}^{m_i}\left(\frac{\sigma_{ij}^2}{n_{ij}}\left(1+\left(n_{ij}-1\right)\rho_{i1}\right)+2\sum_{j'>j}^{m_i}\sigma_{ij}^2\rho_{i2}\right)$$
$$= \frac{\sigma_i^2}{m_i n_i}(1+(n_i-1)\rho_{i1}+n_i(m_i-1)\rho_{i2})$$

(6)

The mean outcome of participants in group A in hospital i, $\bar{y}_i^A$ equals the proportion of participants that will get a surgical site infection, $\hat{p}_{Ai}$. The variance of the estimated proportion is equal to $\hat{p}_{Ai}\,(1-\hat{p}_{Ai})$ = $\bar{y}_i^A(1-\bar{y}_i^A)$. The expected proportion of participants with an SSI will be around $\hat{p}_A = 9\%\ (or\ 15\%)$. Due to clustering of the participants it is realistic that the variance of $\bar{y}_i^A$ will be actually greater. Therefore variance $Var\ (\bar{y}_i^A) = \hat{p}_A\left(1-\hat{p}_A\right)+\tau^2$, τ^2 represents the difference between trial sites. The variance of $\bar{y}_i^B$ will be comparable (with possibly different numbers for $\sigma_i^2,\ \rho_{i1},\ \rho_{i2}$).

However, following the null hypothesis the proportions of group A and group B are not different and $\sigma_i^2,\ \rho_{i1},\ \rho_{i2}$ are then also the same for group A and B.

The final term in (4), $Cov(\bar{y}_i^A, \bar{y}_i^B)$ represents the covariance of the outcomes between group A and group B at a specific trial site. If we assume the following: (v) variance of the outcome is equal for group A and B (this seems reasonable regarding the null hypothesis), (vi) the correlation (ρ_{i3}) between the outcome of participants in group A and B within the same trial site is equal for two participants that received treatment on the same day and (vii) the number of days the randomisation resulted in group A and B are equal;

$$Cov(\bar{y}_i^A, \bar{y}_i^B) = Cov(\tfrac{1}{m_i}\textstyle\sum_j^{m_i}\tfrac{1}{n_{ij}}\sum_\ell^{n_{ij}} y_{ij\ell}, \tfrac{1}{m_i}\sum_{j'}^{m_i}\tfrac{1}{n_{ij'}}\sum_{\ell'}^{n_{ij'}} y_{ij'\ell'}) = \sigma_i^2\rho_{i3}$$

Putting these equations together and taking assumptions i-vii into account, we find;

$$Var(\bar{y}_i^A - \bar{y}_i^B) = \frac{2\sigma_i^2}{m_i n_i}(1+(n_i-1)\rho_{i1}+n_i(m_i-1)\rho_{i2} - m_i n_i \rho_{i3})$$

(7)

Also, if we assume that (viii) the correlation between the outcomes of participants that are randomised for the same day (ρ_{i1}) is equal to the correlation of outcomes of participants that are randomised for a different day (ρ_{i2}) and is equal for both intervention groups (ρ_{i3}), than $\rho_{i1} = \rho_{i2} =$

$\rho_{i3} = \rho_i$, then $Var(\bar{y}_i^A - \bar{y}_i^B) = \frac{2\sigma_i^2}{m_i n_i}(1-\rho_i)$. Lastly (ix) if these variances and correlations are equal (i.e. $\sigma_i^2 = \sigma^2$ and $\rho_i = \rho$) for all k trial sites and (x) the number of days randomisations $m_i = m$ and $n_i = n$ then;

$$Var(\bar{\bar{d}}) = \frac{2\sigma^2}{kmn}(1-\rho) \tag{8}$$

This seems a compact expression for the variance but many assumptions are required.

Example

If the observed effect equals the assumed effect used for the sample size calculation, the event-rate will decrease from 9.10% to 6.37% $\bar{\bar{d}}$ will be 0.0273. The variance σ^2 will be $0.09 * (1-0.09) = 0.08109$. Actually, due to τ (the difference between trial sites), it will be a little higher such as σ^2 = 0.10. If there will be k = 10 trial sites, all participating for 30 days in both group *A* and group *B*, m = 30, with 5 participants per day and $\rho = 0.10$

$Var(\bar{\bar{d}}) = \frac{2\sigma^2}{kmn}(1-\rho) = \frac{2*0.10}{10*30*5}(1-0.10) = 0.00012$

The value of the z-test will be $\frac{0.027}{\sqrt{0.00012}} = 2.467$

If we assume a significance level of $\alpha = 0.05, z_\alpha = 1.96$ we find $z_\beta = 2.46 - 1.96 = 0.50$. The power is $\varphi(0.50) = 0.69$ or 69%. Here $\varphi(\dots)$ represents cumulative standard normal distribution. If all hospitals randomise during $m = 60$ days both interventions, the power will increase to 94%.

It may be realistic to assume that $\rho_3 \le \rho_2 \le \rho_1$, e.g. $\rho_3 = 0,\ \rho_2 = 0.05, \rho_1 = 0.10$ and then the power is only 12% or 13% with $m = 30$ or $m = 60$ respectively. If ρ_3 decreases this will especially lead to a loss in power. As (7) demonstrates $Var(\bar{\bar{d}})$ decreases as ρ_3 increases.

2. Difference between m_i of participating hospitals

In the previously mentioned elaboration, we assumed that the number of randomised days is equal for all hospitals. However this seems not very likely. From (7) $Var(\bar{\bar{d}})$ can be expressed, under the assumptions that $\sigma_i = \sigma,\ \rho_{i1} = \rho_1,\ \rho_{i2} = \rho_2,\ \rho_{i3} = \rho_3$ and $n_i = n$ for all hospitals $i = 1, \dots, k$, as:

$$Var(\bar{\bar{d}}) = \frac{2\sigma^2(\rho_2-\rho_3)}{k} + \frac{2\sigma^2}{k}\left(\frac{1-\rho_1}{n} + (\rho_1-\rho_2)\right)\left(\frac{1}{k}\sum_{i=1}^{k}\frac{1}{m_i}\right) \tag{9}$$

The last term $\frac{1}{k}\sum_{i=1}^{k}\frac{1}{m_i}$ is the inverse of the harmonic mean of the number of days the hospitals randomise participants. If all hospitals contribute the same number of days, this term reduces to only $\frac{1}{m}$, if this number of days the hospitals contribute varies greatly this term $\frac{1}{k}\sum_{i=1}^{k}\frac{1}{m_i}$ will be greater than $\frac{1}{m}$. Consequently the $Var(\bar{\bar{d}})$ will be greater. This seems logical as the mean effect of the intervention $\bar{\bar{d}}$ will be the average of the effects per hospital $\bar{y}_i^A - \bar{y}_i^B$ and the uncertainty of these combines to the uncertainty of $\bar{\bar{d}}$.

NB: (9) also demonstrates that it is expected that $\rho_3 \leq \rho_2 \leq \rho_1$ as otherwise $Var(\bar{\bar{d}})$ could become negative.

If m_i greatly varies, hospitals with a small m will strongly contribute to $Var(\bar{\bar{d}})$. One way to overcome this would be to set m to a minimum and exclude hospitals with m smaller than this minimum. However we do not deem this ethical nor proper scientific conduct. An alternative is to analyse $\bar{\bar{d}}$ in a meta-analytical way.

$$\bar{\bar{d}}^* = \frac{\sum_{i=1}^{k}\frac{(\bar{y}_i^A - \bar{y}_i^B)}{Var(\bar{y}_i^A - \bar{y}_i^B)}}{\sum_{i=1}^{k}\frac{1}{Var(\bar{y}_i^A - \bar{y}_i^B)}} \tag{10}$$

And its variance

$$Var\left(\bar{\bar{d}}^*\right) = \frac{1}{\sum_{i=1}^{k}\frac{1}{Var(\bar{y}_i^A - \bar{y}_i^B)}} \tag{11}$$

If $m_i = m$ for all hospitals, the last formula will reduce to (1), but if m_i is not constant, hospitals with a small m will contribute less to the uncertainty of $\bar{\bar{d}}^*$ and therefore the exclusion of hospitals with a small m will not result in a smaller $Var(\bar{\bar{d}}^*)$

Appendix 3: Completed checklist for Guideline for Statistical Analysis Plan

Table. SAP Guidance Document: Recommended Items to Address in a Clinical Trial SAP[a]

Section/Item	Index	Description	Page
**Section 1: Administrative Information**			
Title and trial registration	1a	Descriptive title that matches the protocol, with SAP either as a forerunner or subtitle, and trial acronym (if applicable)	1
	1b	Trial registration number	3
SAP version	2	SAP version number with dates	3
Protocol version	3	Reference to version of protocol being used	3
SAP revisions	4a	SAP revision history	17
	4b	Justification for each SAP revision	n.a.
	4c	Timing of SAP revisions in relation to interim analyses, etc	n.a.
Roles and responsibility	5	Names, affiliations, and roles of SAP contributors	1
Signatures of:	6a	Person writing the SAP	17
	6b	Senior statistician responsible	17
	6c	Chief investigator/clinical lead	17
**Section 2: Introduction**			
Background and rationale	7	Synopsis of trial background and rationale including a brief description of research question and brief justification for undertaking the trial	4
Objectives	8	Description of specific objectives or hypotheses	4
**Section 3: Study Methods**			
Trial design	9	Brief description of trial design including type of trial (eg, parallel group, multiarm, crossover, factorial) and allocation ratio and may include brief description of interventions	4
Randomization	10	Randomization details, eg, whether any minimization or stratification occurred (including stratifying factors used or the location of that information if it is not held within the SAP)	4
Sample size	11	Full sample size calculation or reference to sample size calculation in protocol (instead of replication in SAP)	4
Framework	12	Superiority, equivalence, or noninferiority hypothesis testing framework, including which comparisons will be presented on this basis	4
Statistical interim analyses and stopping guidance	13a	Information on interim analyses specifying what interim analyses will be carried out and listing of time points	5
	13b	Any planned adjustment of the significance level due to interim analysis	n.a.
	13c	Details of guidelines for stopping the trial early	n.a.
Timing of final analysis	14	Timing of final analysis, eg, all outcomes analyzed collectively or timing stratified by planned length of follow-up	5
Timing of outcome assessments	15	Time points at which the outcomes are measured including visit "windows"	5

			Page
**Section 4: Statistical Principles**			
Confidence intervals and *P* values	16	Level of statistical significance	6
	17	Description and rationale for any adjustment for multiplicity and, if so, detailing how the type 1 error is to be controlled	6
	18	Confidence intervals to be reported	6
Adherence and protocol deviations	19a	Definition of adherence to the intervention and how this is assessed including extent of exposure	6
	19b	Description of how adherence to the intervention will be presented	6
	19c	Definition of protocol deviations for the trial	6
	19d	Description of which protocol deviations will be summarized	7
Analysis populations	20	Definition of analysis populations, eg, intention to treat, per protocol, complete case, safety	7
**Section 5: Trial Population**			
Screening data	21	Reporting of screening data (if collected) to describe representativeness of trial sample	7<br>7
Eligibility	22	Summary of eligibility criteria	7-8
Recruitment	23	Information to be included in the CONSORT flow diagram	8
Withdrawal/follow-up	24a	Level of withdrawal, eg, from intervention and/or from follow-up	8
	24b	Timing of withdrawal/lost to follow-up data	8
	24c	Reasons and details of how withdrawal/lost to follow-up data will be presented	8
Baseline patient characteristics	25a	List of baseline characteristics to be summarized	8
	25b	Details of how baseline characteristics will be descriptively summarized	8
**Section 6: Analysis**			
Outcome definitions		List and describe each primary and secondary outcome including details of:	
	26a	specification of outcomes and timings. If applicable include the order of importance of primary or key secondary end points (eg, order in which they will be tested)	5
	26b	specific measurement and units (eg, glucose control, hbA$_{1c}$ [mmol/mol or %])	5
	26c	any calculation or transformation used to derive the outcome (eg, change from baseline, QoL score, time to event, logarithm, etc)	5
Analysis methods	27a	what analysis method will be used and how the treatment effects will be presented	10
	27b	any adjustment for covariates	12
	27c	methods used for assumptions to be checked for statistical methods	12
	27d	details of alternative methods to be used if distributional assumptions do not hold, eg, normality, proportional hazards, etc	12
	27e	any planned sensitivity analyses for each outcome where applicable	12
	27f	any planned subgroup analyses for each outcome including how subgroups are defined	12
Missing data	28	Reporting and assumptions/statistical methods to handle missing data (eg, multiple imputation)	14
Additional analyses	29	Details of any additional statistical analyses required, eg, complier-average causal effect[10] analysis	12
Harms	30	Sufficient detail on summarizing safety data, eg, information on severity, expectedness, and causality; details of how adverse events are coded or categorized; how adverse event data will be analyzed, ie, grade 3/4 only, incidence case analysis, intervention emergent analysis	15
Statistical software	31	Details of statistical packages to be used to carry out analyses	15
References	32a	References to be provided for nonstandard statistical methods	12
	32b	Reference to Data Management Plan	17
	32c	Reference to the Trial Master File and Statistical Master File	17
	32d	Reference to other standard operating procedures or documents to be adhered to	17

Abbreviations: CONSORT, Consolidated Standards of Reporting Trials; hbA$_{1c}$, hemoglobin A$_{1c}$; QoL, quality of life; SAP, statistical analysis plan.

[a] Reproduced with permission from the authors.

Gamble C, Krishan A, Stocken D, et al. Guidelines for the Content of Statistical Analysis Plans in Clinical Trials. *JAMA.* 2017;318(23):2337–2343. doi:10.1001/jama.2017.18556

Appendix 4: Signature Sheet

Name	Signature	Date
**Author SAP**<br>S.W. de Jonge		01/09/2020
**Author SAP**<br>N. Wolfhagen		12/08/2020
**Senior statistician**<br>A.H. Zwinderman		22/09/2020
**Senior statistician responsible**<br>M.G.W. Dijkgraaf		07/09/2020
**Principle Investigator**<br>M.W. Hollmann		12/8/20
**Principle Investigator**<br>M.A. Boermeester		03/09/2020
