# Supplementary material for: Enhanced PeriOperative Care and Health protection programme for the prevention of surgical site infections after elective abdominal surgery (EPO2CH): statistical analysis plan of a randomised controlled multicentre superiority trial
Source: Trials. 2021 Apr 21;22:297. doi: 10.1186/s13063-021-05202-y (PMC8059309; doi:10.1186/s13063-021-05202-y)
Supplement: Supplementary file 2 — Additional file 2. [file 13063_2021_5202_MOESM2_ESM.pdf]

Appendix 3: Signature Sheet

Name	Signature	Date
**Author SAP**<br>S.W. de Jonge		01/09/2020
**Author SAP**<br>N. Wolfhagen		12/08/2020
**Senior statistician**<br>A.H. Zwinderman		22/09/2020
**Senior statistician responsible**<br>M.G.W. Dijkgraaf		07/09/2020
**Principle Investigator**<br>M.W. Hollmann		12/8/20
**Principle Investigator**<br>M.A. Boermeester		03/09/2020
